# Geographical Variations in Host Predisposition to COVID-19 Related Anosmia, Ageusia, and Neurological Syndromes

**DOI:** 10.3389/fmed.2021.661359

**Published:** 2021-04-29

**Authors:** A Aravin Kumar, Sean Wei Yee Lee, Christine Lock, Nicole CH Keong

**Affiliations:** ^1^Department of Neurosurgery, National Neuroscience Institute, Singapore, Singapore; ^2^Tan Tock Seng Hospital, Singapore, Singapore; ^3^Duke-NUS Medical School, Singapore, Singapore

**Keywords:** anosmia, ageusia, olfactory and gustatory dysfunctions, COVID-19, geographical variations, socio-economic variations, ethnic variations

## Abstract

The novel coronavirus disease (COVID-19), has become the most critical global health challenge in recent history. With SARS-CoV-2 infection, there was an unexpectedly high and specific prevalence of olfactory and taste disorders (OTDs). These high rates of hyposmia and hypogeusia, initially reported as up to 89% in European case series, led to the global inclusion of loss of taste and/or smell as a distinctive feature of COVID-19. However, there is emerging evidence that there are striking differences in the rates of OTDs in East Asian countries where the disease first emerged, as compared to Western countries (15.8 vs. 60.9%, *p*-value < 0.01). This may be driven by either variations in SARS-CoV-2 subtypes presenting to different global populations or genotypic differences in hosts which alter the predisposition of these different populations to the neuroinvasiveness of SARS-CoV-2. We also found that rates of OTDs were significantly higher in objective testing for OTDs as compared to subjective testing (73.6 vs. 60.8%, *p*-value = 0.03), which is the methodology employed by most studies. Concurrently, it has also become evident that racial minorities across geographically disparate world populations suffer from disproportionately higher rates of COVID-19 infection and mortality. In this mini review, we aim to delineate and explore the varying rates of olfactory and taste disorders amongst COVID-19 patients, by focusing on their underlying geographical, testing, ethnic and socioeconomic differences. We examine the current literature for evidence of differences in the olfactory and gustatory manifestations of COVID-19 and discuss current pathophysiological hypotheses for such differences.

## Introduction

The novel severe acute respiratory syndrome coronavirus 2 (SARS-CoV-2) and the resultant coronavirus disease 2019 (COVID-19) is the largest pandemic in recent history. As of 24th January 2021, there have been 96, 877, 399 confirmed cases and 2, 098, 879 confirmed deaths in 224 countries and territories, according to the World Health Organization (WHO). The first cases of COVID-19 were described in Wuhan, China, in late 2019 (1), with initial presenting complaints related to acute respiratory illnesses (ARI) (2–4). However, as the pandemic developed, relatively minor symptoms such as anosmia and ageusia were discovered to be disproportionately important to the presentation and understanding of COVID-19 pathophysiology.

Olfactory and taste disorders (OTDs) were first described in February 2020 by Mao et al. (5) in their retrospective case series describing neurological manifestations amongst COVID-19 patients in Wuhan, China. Out of 314 patients, they reported 5.1% hyposmia and 5.6% hypogeusia (5). As the pandemic spread to Europe, media and anecdotal accounts from medical practitioners supported such reports of OTDs (6). In early April, Lechien et al. (7) published a multicentre cross-sectional study based in several European countries, with 417 patients, of which 85.6 and 88.8% were found to have olfactory dysfunction and gustatory dysfunction, respectively. Shortly after this, multiple otolaryngology chapters released statements recommending that OTDs be considered as symptoms of COVID-19 (8–10). This was followed by the Centers for Disease Control and Prevention (CDC), United States of America (USA), and the Ministry of Health, Singapore adding “loss of smell or taste” to the list of symptoms of COVID-19 in mid-April. The World Health Organization and the Department of Health and Social Care, United Kingdom (UK), officially added “loss of taste or smell” to their respective list of symptoms of COVID-19 in early May.

Anosmia, ageusia and the entire spectrum of OTDs are of importance to our understanding of COVID-19 because they provide an opportunity to learn more about the neurotropic effects of the SARS-CoV-2 virus and allow us to study the potential long-term neurological effects that SARS-CoV-2 infection can lead to, even in patients with mild COVID-19 infections. It is interesting to note, that despite the initial surge of COVID-19 cases in Asia, the literature highlighting OTDs was primarily based on patients in Europe and the USA.

In this mini review we explore the different possible reasons behind these geographical differences in OTD rates, such as the initial stress on Asian healthcare systems, different viral genotypes and differing pathogenic susceptibility of different populations. We also examine variations seen in OTD rates in studies utilizing subjective testing as compared to objective testing. We describe the differences seen between different ethnic groups and explore if genetic determinants can account for the disproportionate affliction of minority races, and other factors such as comorbidity burden and socio-economic status. We also highlight developing trends such as the gender differences in anosmia and ageusia as well as the use of real-time trackers.

## Methodology

We performed searches for studies examining olfactory and gustatory dysfunction amongst COVID-19 patients in databases such as PubMed, Google Scholar and Web of Science. In view of the time-sensitive nature of the COVID-19 pandemic, preprint databases such as Medrxiv and Biorxiv were also utilized to capture latest developments. Search terms utilized included “Anosmia in COVID-19,” “Ageusia in COVID-19,” “Olfactory disorders in COVID-19,” “Gustatory disorders in COVID-19” and other related search terms. Original studies, commentaries and review articles were considered during the literature review. Studies with original data on OTDs were included for comparison and analysis, with the original reported rates of OTDs reflected without any secondary analysis. Pooled averages were calculated for comparison between different geographical regions. Statistical analysis was carried out by SPSS version 20.0 (SPSS, IBM Corporation, IL, USA), and Pearsons Chi-square tests were performed, with *p* < 0.05 regarded as statistically significant.

## Hypothesized Pathophysiological Processes for the Development of Anosmia and Ageusia

SARS-CoV-2 is closely related to severe acute respiratory syndrome coronavirus (SARS-CoV) and the Middle East respiratory syndrome coronavirus (MERS-CoV) – which have each caused their own epidemics associated with extrapulmonary manifestations and high mortality rates (11, 12). The functional receptor allowing for SARS-CoV-2 entry into host cells is human angiotensin-converting enzyme 2 (ACE2) (13), and this viral entry is facilitated by transmembrane protease serine 2 (TMPRSS2), similar to SARS-CoV (14, 15). ACE2 is found in the human airway epithelia, lung parenchyma, vascular endothelia, kidney cells and small intestine cells (16, 17).

SARS-CoV-2 is postulated to be able to infect the CNS in a similar manner to SARS-CoV, via a hematogenous and trans-neuronal route, with cell entry mediated by ACE2 receptors (18). SARS-CoV-2 in the bloodstream may interact with ACE2 expressed in the capillary endothelium of cerebral vessels, and allow viral access to the brain, after which the virus can interact with ACE2 receptors expressed in neurons (18). Viral interaction with the olfactory bulb and cortex may lead to neuronal damage and resultant hyposmia or anosmia (18–21). The trans-neuronal spread of the virus has also been hypothesized to damage the peripheral neurons directly (18, 22, 23). However, olfactory neurons do not express significant levels of ACE2 and TMPRSS2 (24–27) and neuronal damage to the olfactory bulb and cortex cannot account for case reports of rapid and transient anosmia (7), in view of such damage requiring significant time for recovery (27).

Another proposed mechanism for anosmia is damage to non-neuronal structures that support olfactory function, such as olfactory epithelium sustentacular cells, microvillar cells, Bowman's gland cells, horizontal basal cells and olfactory bulb pericytes (25). These olfactory epithelium sustentacular cells have abundant expression of ACE2 and TMPRSS2 (24, 25, 28, 29). Local infection of these non-neuronal structures is proposed to cause significant inflammatory responses affecting olfactory sensory neurons or olfactory bulb neurons, and may even result in neuronal death (25). Reports of transient anosmia, with rapid recovery, may then be explained by the faster regeneration rate of sustentacular cells, as compared to olfactory neurons (20, 27).

Regarding ageusia, ACE2 receptors are known to diffusely express on the mucous membranes of the oral cavity, with a high concentration on the tongue (30). It is thought that ACE2 modulates taste perception, and that SARS-CoV-2 binding to the receptor may lead to taste dysfunction by damaging the gustatory cells, even though the exact mechanism is unclear (31, 32). One proposed mechanism is the binding of SARS-CoV-2 to sialic acid receptors, an ability it shares with MERS-CoV (33). This binding of SARS-CoV-2 to sialic acid receptors may result in the acceleration of degradation of gustatory particles, resulting in blunting of the patient's taste (31). Another possibility is that ageusia happens concomitantly with anosmia due to the close functional correlations between the olfactory and gustatory chemosensory systems (34).

Emerging evidence on neuroimaging characteristics of anosmic patients may also assist to elucidate the definite pathophysiology of COVID-19 associated OTDs. Magnetic Resonance Imaging of COVID-19 patients with OTDs have shown olfactory bulb injury (19) and changes (35–37), suggesting the viral invasion of these nerve structures with resultant sensorineural dysfunction. The persistence of OTDs is also an area of interest, with studies suggesting a persistence of symptoms in up to 24% of COVID-19 patients (38, 39), and interesting trends such as younger patients and female patients having a higher tendency for such persistence (40). It may be only possible with time to elucidate the exact pathophysiological elements leading to OTDs in the context of SARS-CoV-2 infection, and histological biopsies of COVID-19 patients are likely to greatly aid this effort (27).

## Geographical Variations

Anosmia, ageusia and OTDs amongst COVID-19 patients were first recognized to be common in Europe, several months after the first few COVID-19 epicenters in Asia. Asian studies were consistently publishing lower percentages of patients presenting with anosmia and ageusia compared to those being reported in Europe and the USA, with one study reporting the prevalence of chemosensory dysfunction in Caucasians to be three times higher than that in Asians (27, 41–44). Table 1 illustrates the difference in pooled average prevalence of olfactory disorders, taste disorders and combined olfactory and/or taste disorders between Eastern and Western populations, with a map graphically representing the higher prevalence of OTDs in Western countries. The pooled average for the Western population was close to 4 times that of the Eastern populations (15.8 vs. 60.9%, *p*-value < 0.01) as seen in Table 1. Three main reasons have been postulated in the literature with regards to this difference between Western populations and Eastern populations.

**Table 1:** Geographical and testing variations.

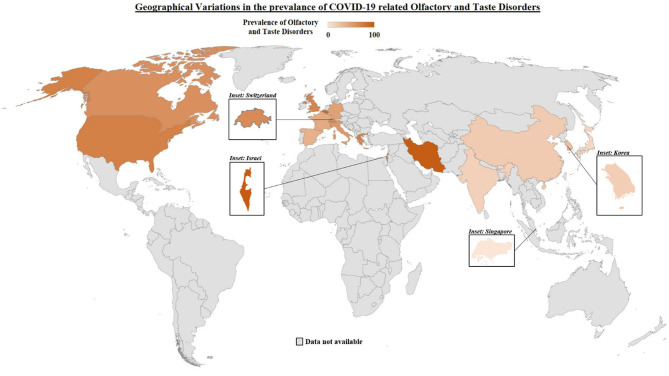							
World Map of the prevalence rates of Olfactory and Taste Disorders globally.							
*(The intensity of color suggests a higher prevalence of OTDs.)*							
**S/N**	**Author**	**Country**	**Sample Size**	**Olfactory and taste assessment method**	**Olfactory disorders only (%)**	**Taste disorders only (%)**	**Any olfactory and/or taste disorders (%)**
***Asian (ex-Middle East) studies***							
1	Mao et al. (5)	China	214	Subjective	5.1	5.6	NA
2	Lee et al. (40)	Korea	3191	Subjective	4.2	3.1	15.3
3	Wee et al. (45)	Singapore	154	Subjective	NA	NA	22.7
4	Chua et al. (46)	Singapore	31	Subjective	22.6	NA	NA
5	Qiu et al. (47)	China	239	Subjective	20.0	3.0	32.0
6	Kim et al. (48)	Korea	172	Subjective	39.5	33.7	NA
7	Komagamine et al. (49)	Japan	628	Subjective	10.0	9.1	NA
8	Mishra et al. (50)	India	74	Subjective	14.8	NA	NA
9	Tham et al. (51)	Singapore	1065	Subjective	11.8	4.6	12.6
**Pooled averages**					**8.3**	**5.1**	**15.8**
***Middle Eastern and Western studies***							
10	Hopkins et al. (6)	England	382	Subjective	60.0	88.9	NA
11	Giacomelli et al. (52)	Italy	59	Subjective	5.1	10.2	33.9
12	Yan et al. (53)	USA	128	Subjective	68.0	71.0	NA
13	Levinson et al. (54)	Israel	45	Subjective	35.7	33.3	69.0
14	Menni et al. (55)	England	579	Subjective	NA	NA	59.0
15	Spinato et al. (56)	Italy	283	Subjective	NA	NA	64.4
16	Klopfenstein et al. (57)	France	114	Subjective	47.0	40.3	Nil
17	Beltran et al. (58)	Spain	79	Subjective	45.2	45.2	39.2
18	Menni et al. (59)	England	7178	Subjective	NA	NA	65.0
19	Zens et al. (60)	Germany	65	Subjective	47.6	NA	NA
20	Patel et al. (61)	England	141	Subjective	56.7	63.1	NA
21	Luers et al. (62)	Germany	72	Subjective	74.0	69.0	68.0
22	Bertlich et al. (63)	Germany	47	Subjective	31.9	19.1	NA
23	Haehner et al. (64)	Germany	69	Subjective	31.8	NA	NA
24	Borobia et al. (65)	Spain	2226	Subjective	12.8	NA	NA
25	Tudrej et al. (66)	France	816	Subjective	19.1	23.0	29.7
26	Qiu et al. (47)	Germany	39	Subjective	18.0	3.0	69.0
27	Qiu et al. (47)	France	116	Subjective	6.0	NA	49.0
28	Gelardi et al. (67)	Italy	72	Subjective	11.0	25.0	47.0
29	Speth et al. (68)	Switzerland	103	Subjective	61.2	65.0	NA
30	Carignan et al. (69)	Canada	134	Subjective	51.5	63.4	64.9
31	Abalo-Lojo et al. (32)	Spain	131	Subjective	NA	NA	55.0
32	Lee et al. (70)	Canada	56	Subjective	42.9	57.1	NA
**Pooled averages (Subjective only)**					**26.1**	**46.9**	**60.8**
33	Kaye et al. (71)	USA	237	Objective	73.0	NA	NA
34	Moein et al. (72)	Iran	60	Objective	98.0	NA	NA
35	Hornuss et al. (73)	Germany	45	Objective	40.0	NA	NA
36	Lechien et al. (7)	Europe	417	Objective	85.6	88.8	NA
37	Vaira et al. (74)	Italy	72	Objective	14.4	12.5	73.6
38	Tsivgoulis et al. (75)	Greece	22	Objective	72.0	NA	NA
**Pooled averages (Objective only)**					**74.2**	**77.5**	**73.6**
*p-values (Subjective vs. objective)*					*<0.01*	*<0.01*	*0.03*
**Pooled averages**					**33.4**	**52.3**	**60.9**
*p-values (Asian vs. Western)*					*<0.01*	*<0.01*	*<0.01*

Firstly, there was the shock element of the initial outbreak. In the initial stages of the outbreak, when it was first recognized in Asia, patients who were critically ill would have been prioritized and hospitalized. Indeed, the literature from the early days of the pandemic highlighted concerns regarding mortality and need for intensive care therapy (76, 77), suggesting that the patients presenting to the healthcare institutions were indeed more unwell. It has been suggested that minor symptoms such as anosmia and ageusia may have been overlooked in preliminary cohorts in the pandemic, both by medical professionals, as well as patients themselves (7, 43). This could have led to an under-reporting of actual anosmia and ageusia rates in Asian countries in the initial stages of the outbreak. However, over time, this has become a less viable explanation, in view of studies from other Asian countries also showing significantly lower rates of anosmia and ageusia as compared to Western nations (40, 45).

The second possible reason is that of differing viral genotypes in Asia as compared to Europe and the USA. A phylogenetic analysis of 160 SARS-CoV-2 genomes by Forster et al. (78) found 3 central variants of the virus: Types A, B and C. Types A and C were found to be more prevalent amongst Europeans and Americans, compared to Type B which was more prevalent amongst Asians (78). Types A and C are speculated to have high pathogenicity for the nasal cavity, hence resulting in the higher prevalence of olfactory and taste disorders in Western populations (43, 78). Mutations in the receptor binding domain (RBD) of the virus spike protein (subunit S1) may also result in differing viral tropism and infectivity (79). Mutations in the RBD have been shown to affect its binding to the ACE2 receptor (80, 81), and these mutations can impact the pathogenicity of the virus (82). Indeed, early studies probing interactions between ACE2 coding variants and SARS-CoV-2 virus have pointed to certain populations having a higher predisposition for SARS-CoV-2 binding (83). The emergence of new variants such as the UK variant (84) and South African variant (85) in late 2020 and early 2021 lend further credence to the presence of differing viral genotypes in distinct geographical territories. These variants may have differing rates of infectivity of the olfactory epithelium which may influence the prevalence of OTDs (27).

Finally, differing pathogenic susceptibility, in the form of genetic variations of host proteins and receptors such as ACE2 and TMPRSS2, may have led to the difference in anosmia and ageusia rates between different populations. Variations in ACE2 expression in different populations have been reported (86, 87), with one study finding increased ACE2 expression in tissues in East Asian populations (88). Variations in TMPRSS2 protein frequency have also been observed with European populations having much higher levels of pulmonary expression as compared to East Asian populations (20). Genetic differences in ACE2 variants, characterized by post-translational modifications such as glycosylation, may also contribute to the varying susceptibility of different populations to anosmia (27, 89). Such genetic differences resulting in differing OTD rates were corroborated by a Singaporean study, which collected nationality and ethnicity data, and found that Caucasians were 3.05 times more likely to have OTDs as compared to Chinese, South East Asian and West Asian races (51). Further research is required to delineate the link between ACE2/TMPRSS2 expression and susceptibility to olfactory and taste disorders.

The high susceptibility to OTDs amongst Western populations as compared to East Asian populations, raises the specter of whether these same Western populations are facing a higher burden of SARS-CoV-2 related peripheral and central nervous system disorders. The same reasons of possibly different viral genotypes and differing pathogenic susceptibility can also be used to explain any corresponding spike in both PNS and CNS manifestations in Western populations as compared to Eastern populations. We should note that directly comparing prevalence of neurological symptoms between studies has proven to be difficult, largely due to the heterogenous nature of recorded neurological symptoms such as headache, giddiness and altered mental state – especially as they may be manifestations of systemic disease as well (90, 91). Nevertheless, comparing CNS syndromes, such as encephalitis, and PNS syndromes, such as mono or polyneuropathies, reveals no evidence of increased rates of such syndromes amongst Western populations compared to Eastern populations thus far (92, 93).

## Testing Variations

The majority of the literature concerning COVID-19 and OTDs has been based on patient self-reporting (94). This may inevitably lead to inconsistences (52, 94), such as recall bias on the part of the patient, or confirmation bias on the part of the medical professional. Objective forms of testing have been proposed and utilized in some studies, such as the University of Pennsylvania Smell Identification Test (UPSIT), Questionnaire of Olfactory Disorders–Negative Statements (95, 96), COVID-19 Anosmia Reporting Tool (71), Sniffin' sticks test, and Korean version of Sniffin' sticks test (KVSS) (97). Broadly, studies utilizing objective testing for anosmia and ageusia have found a higher prevalence of olfactory and gustatory disturbances amongst COVID-19 patients (72, 98). Table 1 highlights the differences in OTD rates between objective and subjective testing, seen in the differing prevalence rates, in favor of objective testing (60.8 vs. 73.6%, *p*-value = 0.03). We can hypothesize that the reasons behind under-reporting of anosmia or ageusia may be due to difficulties in perceiving a reduction in sense of smell or taste (99) as well as difficulties in finding and receiving an appropriate level of care (100), which may be linked to socio-economic issues, further elaborated on below. It has to be appreciated however, that self-reporting of symptoms may often be the only feasible and practical way of data collection, especially with pandemic precautions and restrictions (44).

## Ethnic, Comorbidity and Socio-Economic Variations

COVID-19 has disproportionately affected racial minorities across the world, with infection rates and mortality rates two to three times higher in these minorities than their proportion in the population (101–106). Ethnic, socio-economic and comorbidity variations all have a role in accounting for this higher affliction rate amongst racial minorities (105).

Variations in OTDs, due to COVID-19, between different ethnicities residing in the same region, have yet to be described fully in the literature. We know from pre-COVID studies that anosmia is more prevalent amongst African-Americans as compared to Caucasians in the USA (107). Dong et al. described the prevalence of anosmia amongst African-Americans as 22.3%, as compared to 10.4% amongst Caucasians, but were unable to account for this stark racial disparity (107). As such, it would not be surprising if anosmia rates in African-American COVID-19 patients were higher than in other ethnicities. A possible explanation may be in the differences in ACE2 expression. A reduced molecular expression of ACE2 in African-descent populations has been described (108), which should theoretically lead to a lower incidence of COVID-19 in these populations, contrary to reality. Vinciguerra et al. proposed that whilst this reduced expression of ACE2 can lead to lower susceptibility to SARS-CoV-2 infection, once infected, the clinical manifestations may be worse, due to progression of inflammatory and thrombotic processes as a result of such reduced ACE2 expression (109). TMPRSS2 may also play a part in the ethnic variations in anosmia. Ethnic differences in TMPRSS2 gene-related activity in prostate tissue have been associated with a higher incidence of prostate cancer in African-American men, as compared to Caucasian men, in the USA (110). This ethnic difference was found to be similar for nasal gene expression of TMPRSS2. In a study of 305 unique nasal epithelial samples, African-Americans were found to have statistically significantly higher TMPRSS2 expression as compared to Asian, Latino, mixed race and Caucasian individuals (111). TMPRSS2 is known to be essential in SARS-CoV-2 cell entry (15), suggesting a possible reason behind the higher burden of COVID-19 infection amongst African-Americans in the USA, possibly holding true for anosmia as well.

Comorbidity burden has been positively correlated with the severity of COVID-19 and mortality (112). This is of particular interest when analysing the impact of COVID-19 on minority races, as comorbidity burdens in ethnic minorities have been found to be higher (101, 105, 113, 114). Several comorbidities such as cardiovascular disease, diabetes, chronic kidney disease and chronic obstructive pulmonary disease have been reported in higher percentages amongst COVID-19 mortality statistics (115). The impact of comorbidities are further highlighted when considering that mortality rates amongst African-American and Caucasian patients in the USA are not significantly different when comorbidities are corrected for (116). However, anosmia tends to affect individuals with fewer comorbidities, except for asthma, which was found to be of a high proportion in patients presenting with anosmia (7, 57). This could possibly be due to anosmia being the only symptom in mild and moderate COVID-19 infections, which tend to occur more often in patients with no or low comorbidity burdens (7). This implies that COVID-19 patients with only isolated OTDs may have a milder disease process.

Possibly the most important piece in explaining the higher proportion of racial minorities being infected with COVID-19 is the socio-economic aspect of the disease. Poverty has been associated with a higher risk of intensive care unit admissions in the USA (117), and a large study in Brazil found that patients from lower socio-economic regions had a higher mortality rate (102). Patients in lower socio-economic regions also have more comorbidities, suggesting that structural health disparities and poor access to healthcare result in poorly controlled chronic diseases (102, 103). People from lower socio-economic classes were also unable to comply with pandemic measures such as social distancing or working from home, due to their crowded living conditions or the blue-collar occupations that many hold (101, 105, 115). In Scotland, COVID-19 patients living in areas with the greatest socio-economic deprivation had a higher frequency of critical care admission and a higher adjusted 30-day mortality, with healthcare facilities in areas with higher socio-economic deprivation also operating at higher occupancy rates (118). The relationship between OTDs and socio-economic status alludes to the differing access to healthcare between different socio-economic groups. A pre-COVID-19 study in South Korea found that high-income population groups had a 1.4 times higher incidence of anosmia as compared to low-income population groups (119). The authors attributed this to the accessibility of medical care to patients with different income levels, and concluded that anosmia can be frequently underestimated by the elderly and low-income due to their economic situation, which hinders them from seeking medical care (119). We can hypothesize that the incidence of OTDs in lower socio-economic groups may be higher in the COVID-19 outbreak, but may not be reflected in the data due to socio-economic factors that hinder their access to healthcare. Future studies on the prevalence of OTDs in different socio-economic groups affected by COVID-19 will help to corroborate this hypothesis.

In addition to the inequalities described above, there may be emerging evidence that gender distinguishes both susceptibility to COVID-19 and associated complications such as anosmia and ageusia (Table 2); further study is required to explain such differences. The ability for public health and research groups to mobilize the efforts of its “citizen scientist” community during this pandemic has also been key to illustrating emerging or unusual trends, such as OTDs, in the form of trackers (Table 2). Despite the limitations of these trackers, they provide both helpful and near real-time updates of disease prevalence as well as gauge societal attitudes toward such group efforts in global health.

**Table 2:** Gender Variations in COVID-19 related olfactory and taste disorders (OTDs) and COVID-19 trackers.

**S/N**	**Author**	**Year**	**Summary/Interpretation**
***Gender Variations in COVID-19 related OTDs***			
1	Lechien et al. (7)	2020	In a study of 417 mild-to-moderate COVID-19 patients, females were found to be significantly more affected by olfactory and taste dysfunctions then males. This was attributed to gender-related differences in inflammatory reaction processes.
2	Hopkins et al. (6)	2020	Online survey of 382 patients reporting self-diagnosed new onset of olfactory and taste dysfunction, of which 74.6% were female. However in view of this being a voluntary online survey, it may simply reflect gender differences in completing such voluntary online questionnaires rather than gender-related differences in prevalence of olfactory and taste dysfunction.
3	Tham et al. (51)	2020	Out of 1065 patients with laboratory-confirmed COVID-19, the female gender was found to be significantly associated with olfactory and taste disorders on multivariate analysis. This was again attributed to gender-related differences in the inflammatory reaction process.
4	Giacomelli et al. (52)	2020	Cross-sectional survey of 59 COVID-19 positive patients of which females were found to have a higher prevalence of olfactory and taste disorders as compared to males (52.6% vs. 25%).
5	Foster et al. (120)	2020	Amongst 949 COVID-19 positive patients, anosmia was significantly associated with younger age, higher BMI as well as female sex. The proportion of females amongst patients with anosmia was significantly higher than that of patients without anosmia (64.7% vs. 52.8%). Anosmia was found to be an independent positive prognostic factor of a less severe COVID-19 infection.
7	Talavera et al. (121)	2020	Amongst 576 COVID-19 positive hospitalized patients, anosmia was present in 25.3%. Patients with anosmia were more frequently female, had less comorbidities such as hypertension and diabetes, and were less likely to be smokers. Hospitalized COVID-19 patients with anosmia had a lower adjusted mortality rate and a less severe course of the disease.
***Proposed mechanisms for gender differences in COVID-19 related OTDs***			
8	Lefevre et al. (122)	2019	Higher levels of inflammatory cytokines were recorded in males as compared to both women and patients with Klinefelter syndrome following whole blood stimulation, even after adjusting for sex steroid levels. This suggests that males may have a more severe disease process as compared to females.
9	Hewegama et al. (123)	2009	In a comparison study of T-cell gene expression between males and females, females were found to have a higher expression of inflammatory and cytotoxic effector molecules under conditions of repeated stimulation. The authors hypothesized that this may contribute to the development and severity of autoimmune diseases in women.
10	Jaillon et al. (124)	2019	Study examining variation in innate immunity, measured by the level of tumor necrosis factor (TNF) in lipopolysaccharide (LPS)-stimulated whole-blood culture found that females have a nearly 30% lower innate immune response.
11	Marriot et al. (125)	2006	Review article outlining differences in innate immune responses between males and females, particularly that viral infections are more severe and require hospitalization more in males than females, corresponding with higher levels of TNF-α in males than females. Females were also found to mount more effective adaptive immune responses to viral pathogens. These favorable differences in innate immune responses are a consequence of higher estrogen levels, which augment immune responses after infection and have been shown to increase resistance to infections.
12	Bwire et al. (126)	2020	Amongst COVID-19 patients, males have been found to have a higher mortality and morbidity. Biological factors such as genetics and immunology play an important role, but the impact of gender behavior cannot be discounted. Males were found to have a higher burden of pre-existing conditions such as diabetes, hypertension and obesity. Males were also found to have higher rates of smoking and alcohol consumption as well as having a tendency to be less likely to comply with preventive measures such as hand washing, stay home orders and donning of face masks. These may all have contributed to the higher morbidity and mortality amongst males compared to females.
13	Kopel et al. (127)	2020	Review article about gender variations in COVID-19 infection. Females are less likely to produce extreme immune responses as compared to males due to X-chromosome and sex hormone modulated innate and adaptive immunity differences. This study also explores gender differences in ACE2 receptor highlighting that ACE2 expression is higher in males than females, but also high in pregnant female patients. This suggests that pregnant female patients may be more susceptible to COVID-19 infection that non-pregnant female patients.
***COVID-19 Trackers***			
14	**COVID-19 Symptom Study** *Menni et al. (59) Drew et al. (128)*	2020	The COVID-19 Symptom Study (previously known as COVID-19 symptom tracker) is a smartphone-based application that was launched in the United Kingdom and United States on March 2020. The application captures self-reported information including age, health risk factors and location. It has registered millions of participants and studies with this dataset found that the proportion of participants who reported olfactory and taste disorders was higher in those with a positive COVID-19 test result compared to those with a negative test result.
15	**COVID-19 Symptom Tracker** *Zens et al. (60)*	2020	The COVID-19 Symptom Tracker is a smartphone-based application, which was designed in Germany and was launched in Germany on April 2020. It captures self-reported demographic and medical history as well as prompting users to report symptoms of COVID-19 on a daily basis. This application registered 11,829 participants who completed the symptom questionnaire at least once, and found that loss of smell was one of the top 5 strongest predictors for COVID-19 infection.
16	**Google Trends analysis** *Walker et al. (129) Cherry et al. (130)*	2020	Analysis of internet search engine interest (Google Trends) for terms relating to olfactory and taste disorders and then correlating them with region specific COVID-19 data. An analysis of such trends found that there was a strong correlation between daily search volumes related to anosmia/ageusia and increases in daily COVID-19 cases and deaths in the same geographical region. Tracking of such search interest can assist public health planning on a regional and/or national level.
17	**COVIDCast** *Flaxman et al. (131)*	2020	COVIDCast is the largest public repository of geographically detailed, real time indicators of COVID-19 activity in the United States of America run by the Delphi lab at Carnegie Mellon University. It gathers data from Facebook via national daily surveys as well as de-identified medical insurance claims. It has garnered more than 15 million responses since starting in April 2020 and has approximately 55,000 participants daily. It does not collect symptom information related to olfactory and taste disorders.
18	**Coronaisrael survey** *Rossman et al. (132)*	2020	Real-time nationwide survey of coronavirus symptoms via an online survey (https://coronaisrael.org/) which is filled out anonymously, collecting primarily geographical data. The survey attained a cumulative number of close to 75,000 responses within 10 days. The newer version of this questionnaire included loss of smell and taste as symptoms of COVID-19 infection. This tracker is a member of the coronavirus census collective.
19	**HowWeFeel** *Segal et al. (133)*	2020	HowWeFeel is a symptom tracker mobile application which administers a 30-second survey on the participants well-being to collect epidemiological data. This data is anonymous and gathers health and demographic data to educate the researchers about infection trends in the community. This tracker is member of the coronavirus census collective.
20	**The Sex, Gender and COVID-19 project** (134)	2020	Live tracking of COVID-19 statistics globally, with a specific focus on sex and gender. As of 24 January 2021, for every 10 Intensive Care Unit (ICU) admissions amongst females, there are approximately 19 ICU admissions amongst males. This tracker does not collect data with regards to olfactory and taste disorders.
21	**CoEpi (Community Epidemiology In Action)** (135)	2020	CoEpi is an open-source mobile application that uses Bluetooth proximity data to anonymously track and alert users who have been in close proximity to symptomatic users. The application captures symptoms related to COVID-19 and other transmissible illnesses. This tracker has yet to publish data which it has collected.
22	**Beat COVID-19 Now** (136)	2020	Beat COVID-19 Now is a symptom tracker mobile application and webpage developed by the Swinburne University of Technology in Australia and captures self-reported COVID-19 symptom information from users worldwide. This tracker has yet to publish data which it has collected.

## Conclusion

Anosmia and ageusia have become well-recognized symptoms of this current pandemic. Much has changed since the original case reports about olfactory and taste disorders, but there are still many questions that remain unanswered regarding how biological and societal factors influence the impact of SARS-CoV-2. In this mini review, we categorize and collate current available literature in order to describe the differences in OTDs seen in different geographical regions as well as amongst different ethnicities and socio-economic conditions. We believe our study to be the first mini review to compare and contrast the variously reported global variations in OTDs. Concurrently, we have provided an up-to-date report on the disproportionate influence of ethnic, comorbidity and socio-economic factors toward such variations. Understanding such inequalities may highlight areas of consideration for allocation of resources and focused attention. Further research is also required to elucidate the exact pathophysiological mechanisms underpinning the phenomena of anosmia and ageusia in COVID-19 and account for other variations, such as the importance of gender toward the clinical phenotype of disease.

## Author Contributions

AK, CL, and NK: conceived of the direction and scope of the review and performed the literature reviews and compilation of references. AK and SL: wrote the manuscript with supervision and direction from CL and NK. All authors contributed to the article and approved the submitted version.

## Conflict of Interest

The authors declare that the research was conducted in the absence of any commercial or financial relationships that could be construed as a potential conflict of interest.
